# Promotion effect of the blend containing 2'-FL, OPN and DHA on oligodendrocyte progenitor cells myelination *in vitro*

**DOI:** 10.3389/fnut.2022.1054431

**Published:** 2022-11-10

**Authors:** Qinggang Xie, Youbo Zhang, Jinlan Zhang, Dongying Cui, Qile Zhou, Mingruo Guo

**Affiliations:** ^1^College of Food Science, Northeast Agricultural University, Harbin, China; ^2^Heilongjiang Feihe Dairy Co., Ltd., Beijing, China; ^3^State Key Laboratory of Natural and Biomimetic Drugs, Department of Natural Medicines, School of Pharmaceutical Sciences, Peking University, Beijing, China; ^4^Beijing Institute of Nutritional Resources, Beijing Academy of Science and Technology, Beijing, China; ^5^Department of Nutrition and Food Science, College of Agriculture and Life Sciences, University of Vermont, Burlington, VT, United States

**Keywords:** myelination, 2'-fucosyllactose, osteopontin, docosahexaenoic acid, neural primary cell

## Abstract

During early neurodevelopment of infant, myelination plays an essential role in brain connectivity and emergence of behavioral and cognitive function. Early life nutrition is an important factor to shape myelination and consequently cognitive appearance. To analyze the effects of additive nutrients, including 2'-fucosyllactose (2'-FL), osteopontin (OPN), docosahexaenoic acid (DHA), on neurocognitive function and brain structure, the current study evaluated the effects of different composition of breast milk nutrients on oligodendrocyte progenitor cells (OPCs) myelination with a neural primary cell model *in vitro*. The study showed that the three nutrients promoted the proliferation, maturation and differentiation of OPCs into mature oligodendrocytes (OLs) in each phage of the cell growth, and the effect of the nutrients blend is obviously stronger than that of the nutrient treatment alone, showing a synergistic effect in promotion of OPCs. The results of this experiment clarified the effects of 2′-FL OPN and DHA to promote myelination development of neural cells, and laid an experimental basis for further optimization of infant formula.

## Introduction

Early life nutrition plays a critical role in neurodevelopmental processes such as neuronal maturation, synaptogenesis, and myelination (1). Myelination is the process of specialized glial cells oligodendrocytes (OLs) in the central nervous system (CNS), to form myelin sheaths around axons, which is essential for normal brain cells connectivity (2, 3). Myelin sheath, composed of several condensed lipid bilayer membranes (4), increases axonal conduction velocity and the maturation of cognitive function by reducing the capacitance of the axonal membrane and allows jumping currents (5–7). Oligodendrocyte progenitor cells (OPCs) are the main glial cell population in the central nervous system, accounting for 5–8% of the total cell population (8). Post-mitotic OPCs differentiate into myelinated OLs, and these OLs expand a number of processes, establishing contacts with axons of different neurons and initiating myelination (9, 10). During their maturation, OLs produce different components of myelin as lipids (cholesterol, galactolipids, and phospholipids) and myelin-specific proteins. The types of myelin proteins expressed by OLs, such as myelin-associated glycoprotein (MAG) and myelin basic protein (MBP), closely correlate with its maturity (11, 12). Myelinated OLs express MAG, and the expression of MAG gradually increases during the maturation of OLs. MAG is a sialic acid-binding immunoglobulin-like lectin, and although it constitutes only a small fraction of the total protein content of myelin, it is predominantly expressed in the peri-axonal region of myelin (13). It appears to play an important role in oligodendrocyte-axon interactions and mediate bidirectional signaling between axons and OLs to support myelination (14). MBP is expressed in mature myelinated OLs and is one of the main components of myelin. MBP appears to play an active role in myelination and compaction. In fact, MBP aggregates and forms a cohesive reticulin network, which is essential for hopping currents (15, 16).

In the CNS, every step of myelination, including the proliferation of OPCs, the differentiation and maturation of OPCs into myelinating OLs, and myelination, is highly regulated by both external and internal factors. In particular, different nutrients have different effects on myelination, suggesting that early life nutrition may have important implications for the regulation of myelination. Therefore, identifying early-life nutritional factors that support myelination is critical for optimal brain and cognitive development.

Osteopontin (OPN), 2′-fucosyllactose (2′-FL), and docosahexaenoic acid (DHA) are essential nutrients in breast milk and infant formula. Many studies have proved that OPN plays an important role in organism, especially in the process of immune activation, bone damage repair, vascular regeneration and bone remodeling (17). 2′-FL, a breast milk oligosaccharide, possesses physiological functionalities of prebiotics effect, antiadhesive antimicrobials, immunomodulation and promotion of brain development (18). DHA is a key nutritional n-3 PUFA and was found to have a strong influence on brain health (19). Though having promotion on neurocognitive function and brain structure, the underlying mechanism of the three essential nutrients on development of neural cells remains unknown by now. In the current study, an *in vitro* model of primary cell cultures containing neurons and OLs was used to evaluate the effects of a composition of breast milk nutrients on myelination. We hoped to add nutrients to mixed cell cultures to promote the proliferation, maturation and differentiation of OPCs into mature OLs and/or the myelinating properties of OLs. The density of OPCs was firstly evaluated after 12 days of *in vitro* culture. Then, OPCs differentiation into OLs and OLs maturation and myelination were assessed by quantifying MAG-positive cells and MBP-positive cells at 18 and 30 days, respectively.

## Methods, materials, and instruments

### Materials and reagents

2′-FL, OPN and DHA were purchased from Beijing Jinkangpu Food Science & Technology Co., Ltd (Beijing, China). Neural cell culture medium was obtained from GibcoTM (Life Technologies Inc., Grand Island, NY, USA). A2B5 antibody (Lot. MAB312RX) and MAG antibody (Lot. MAB1567) were got from Merck Co., Inc., (NJ, USA). MBP antibody (Lot. NBP1-05204) was obtained from Novus Biologicals.

### Neural primary cell acquisition

The animal experiment was approved by the Laboratory Animal Center of Peking University Health Science Center (Beijing, China). To get primary mixed cultures of neurons and OLs (20), forebrains of neonatal rat were taken out on ice and trypsinized for 20 min at 37°C (Trypsin EDTA 1X, PAN BIOTECH). The reaction was stopped by the addition of Dulbecco's modified Eagle's medium (DMEM, PAN BIOTECH) containing DNAase II (0.1 mg/ml, PAN BIOTECH) and 10% fetal bovine serum (FCS, GIBCO). Cells were mechanically dissociated three times by 10 ml pipette and centrifuged at 515 g for 10 min at 4°C, and then were seeded in plates (2 × 10^4^ cells/well) pre-coated with poly-L-lysine (BD Falcon) and laminin (Sigma) in a humidified incubator. The medium consisted of Neurobasal (GIBCO) supplemented with 2% B27 (GIBCO), 2 mM L-glutamine (L-Glu, PAN BIOTECH), 2% P/S solution (PAN BIOTECH), 1% FCS and 10 ng/ml of platelet-derived growth factor (PDGF-AA, PAN BIOTECH).

### Neural cell culture

Cells were seeded in 48-well-plates at a density of 2 × 10^4^ cells/well and added mix or individual nutrients in fresh medium 6 h later. The incubations were replaced with half of the medium containing the same mix or individual nutrients every other day, and stop at 12, 18, or 30 days for immunohistochemistry analysis.

### Immunohistochemistry assay

Immunocytochemistry was carried out as previous report (20) with minor modification. After incubation with the nutrients for 12, 18 and 30 days, the cells were fixed with a cold mixture of 95% ethanol and acetic acid (5%) for 5 min. Non-specific sites were then blocked with 0.1% saponin (Sigma) and 1% FCS (GIBCO) in PBS for 15 min at room temperature.

At 12 days, the cells were incubated with mouse monoclonal anti-A2B5 conjugated Alexa fluor 488 (1/200, MAB312RX) in PBS containing 1% FCS and 0.1% saponin for 2 h at room temperature. After washing with PBS for 3 times, the cells were incubated with a rabbit anti-neurofilament antibody (1/500, N4142) PBS containing 1% FCS and 0.1% saponin for 2 h at room temperature. Neurofilament (NF) was stained with a secondary goat anti-rabbit CF568 antibody (1/400, SAB4600084, SIGMA) containing 1% FCS and 0.1% saponin in PBS for 1 h at room temperature.

At 18 days, the cells were incubated with a mouse monoclonal Anti-MAG (1/400, MAB1567, Millipore) and rabbit anti-NF antibody (1/500, N4142, SIGMA) in PBS containing 1% FCS and 0.1% saponin for 2 h. After washing with PBS for 3 times, the cells were incubated with a secondary goat anti-mouse CF488A antibody (1/400, SAB4600042, SIGMA) and goat anti-rabbit CF 568 antibody (dilution: 1/400, SIGMA, SAB4600084) in PBS containing 1% FCS and 0.1% saponin at room temperature for 1 h. For all conditions, the cell nuclei were stained using a Hoechst solution (SIGMA, B1155).

At 30 days, the cells were incubated with a mouse monoclonal anti-MBP (1/1000, NBP1-05204, NOVUS) and a rabbit anti-Neurofilament antibody (1/500, N4142, SIGMA) in PBS containing 1% FCS and 0.1% saponin for 2 h. After washing with PBS for 3 times, the cells were incubated with goat anti-mouse CF488A antibody (1/800, SAB4600042, SIGMA) and goat anti-rabbit CF568 antibody (1/400, SAB4600084, SIGMA) in PBS containing 1% FCS and 0.1% saponin at room temperature for 1 h.

### Microscopic analysis

Digital images were collected at 20x magnification using ImageXpress equipped with LED lights (excitation 360/480/565 and emission 460/535/620). All images were acquired with the same settings. The number of OPCs was calculated by quantifying the number of A2B5-expressing cells at 12 days, and the results were expressed as the average number of A2B5-expressing cells per well. Differentiation of OPCs to OLs was assessed by counting the number of MAG-positive cells at 18 days. Results are expressed as the average number of cells per well. At 30 days, the maturity of OLs was estimated by counting the number of MBP-positive cells.

### Cell experiment grouping and dosages

To evaluate the function of nutrients in promoting the proliferation, maturation and differentiation of neuronal OPCs into mature OLs and/or myelination of OLs, the effects of different doses of nutrients mixture were studied using the isolated primary neuronal cells (Table 1). The density of OPCs, OPCs differentiation into OLs and OLs maturation, and level of myelination were assessed after 12, 18, and 30 days of *in vitro* culture, respectively.

**Table 1:** Groups and dosages of the nutrition.

**Groups**	**Nutrition composition**	**2-FL(mg/ml)**	**OPN(mg/ml)**	**DHA(mg/ml)**
Control	Vehicle	0	0	0
Positive	Olesoxime	0	0	0
1	2-FL(H^a^)	10	0	0
2	OPN(H^a^)	0	1	0
3	DHA(H^a^)	0	0	5
4	2-FL(L^b^)+OPN(L^b^)	0.1	0.01	0
5	2-FL(L^b^)+OPN(H^a^)	0.1	1	0
6	2-FL(M^c^)+OPN(M^c^)	1	0.1	0
7	2-FL(H^a^)+ OPN(L^b^)	10	0.01	0
8	2-FL(H^a^)+OPN(H^a^)	10	1	0
9	2-FL(LM^d^)+OPN(LM^d^)	0.5	0.05	0
10	2-FL(MH^e^) +OPN(MH^e^)	5	0.5	0
11	2-FL(L^b^)+OPN(L^b^)+DHA(L^b^)	0.1	0.01	0.05
12	2-FL(L^b^)+OPN(M^c^)+DHA(M^c^)	0.1	1	0.5
13	2-FL(H^a^)+OPN(H^a^)+DHA(H^a^)	10	1	5
14	2-FL(LM^d^)+OPN(LM^d^)+DHA(LM^d^)	0.5	0.05	0.25
15	2-FL(MH^e^)+OPN(MH^e^)+DHA(MH^e^)	5	0.5	2.5

a, high dose; b, low dose; c, medium dose; d, low to medium dose; e, medium to high dose.

### Statistical analysis

The results were expressed as mean ± standard error (mean ± SEM), and SPSS software (version 26.0, IBM, Armonk, NY, USA) was used for *T*-test and one-way ANOVA test. A value of *P* < 0.05 was considered statistically significant, while it was judged to be extremely significant when *p* < 0.01.

## Results and discussion

### Pro-proliferative effect of the nutrients on OPCs

To measure the effect of mixed nutrition or nutrient treatment alone on OPCs, the number of A2B5-labeled positive cells were assessed after 12 days to estimate the number of OPCs.

**Figure 1:**
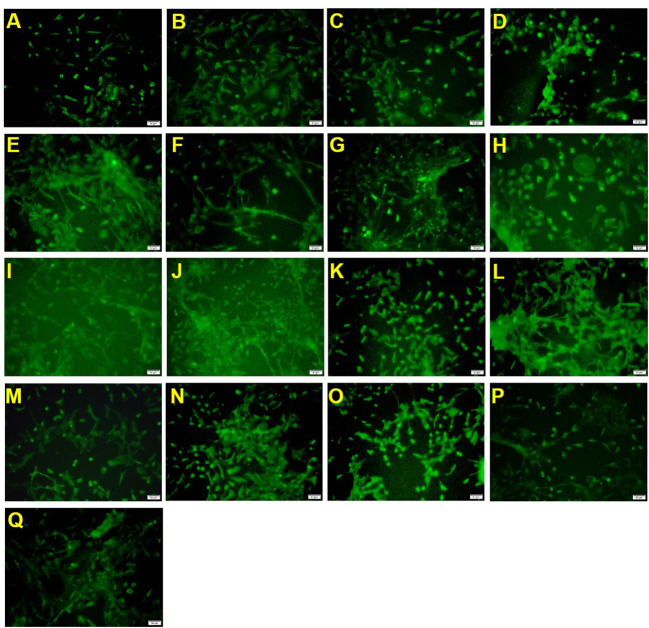
A2B5 immunostaining of neural primary cells after treatment with 2′-FL and OPN and DHA for 12 days. **(A)**, control group; **(B)**, positive drug; **(C)**, 2′-FL(H); **(D)**, OPN(H); **(E)**, DHA(H); **(F)**, 2′-FL(L)+OPN(L); **(G)**, 2′-FL(L)+OPN(H); **(H)**, 2′-FL(M)+OPN(M); **(I)**, 2′-FL(H)+OPN(L); **(J)**, 2′-FL(H)+OPN(H); **(K)**, 2′-FL(LM)+OPN(LM); **(L)**, 2′-FL(MH) +OPN(MH); **(M)**, 2′-FL(L)+OPN(L)+DHA(L); **(N)**, 2′-FL(L)+OPN(M)+DHA(M); **(O)**, 2′-FL(H)+OPN(H)+DHA(H); **(P)**, 2′-FL(LM)+OPN(LM)+DHA(LM); **(Q)**, 2′-FL(MH)+OPN(MH)+DHA(MH). Scale bar: 50 μm.

The sample processing results (Table 2 and Figure 1) showed that the positive drug olesoxime increased the number of A2B5 positive cells compared to the control group. 2′-FL, OPN and DHA high-dose groups (groups 1, 2 and 3) could also significantly increase the number of A2B5 positive cells, indicating that the three components contributed to the proliferation of oligodendrocyte precursor cells. However, the 2′-FL+ OPN and 2′-FL+ OPN + DHA low dose groups (groups 4 and 11) failed to increase the number of A2B5 positive cells, which suggested that the low-dose group set could not effectively induce the proliferation of oligodendrocyte precursor cells. The effects of 2′-FL + OPN middle dose group (group 6), 2′-FL + OPN high dose group (group 8), 2′-FL + OPN middle to high dose group (group 10), and 2′-FL + OPN + DHA high dose group (group 13) and 2′-FL + OPN + DHA medium to high dose group (group 15) were all significantly higher than those of the high dose groups of 2′-FL, OPN or DHA (groups 1, 2 and 3), indicating that these combinations have a synergistic effect on the growth of OPCs.

**Table 2:** Effects of different nutritional compositions containing 2'-FL, OPN and/or DHA on the number of A2B5 positive cells.

**Groups**	**Nutrition composition**	**Density of A2B5 positive cells (Mean ±SEM)**
Control	Vehicle	77.333 ± 5.181
Positive	Olesoxime	113.833 ± 4.708***
1	2-FL(H^a^)	102.167 ± 6.036**
2	OPN(H^a^)	95.50 ± 4.595*
3	DHA(H^a^)	100.333 ± 5.327*
4	2-FL(L^b^)+OPN(L^b^)	85.833 ± 5.009
5	2-FL(L^b^)+OPN(H^a^)	101.00 ± 7.832**
6	2-FL(M^c^)+OPN(M^c^)	114.50 ± 8.563^***∧▽^
7	2-FL(H^a^)+ OPN(L^b^)	110.333 ± 4.256***
8	2-FL(H^a^)+OPN(H^a^)	122.667 ± 5.823^***∧∧▽▽▽^
9	2-FL(LM^d^)+OPN(LM^d^)	108.167 ± 5.023***
10	2-FL(MH^e^)+OPN(MH^e^)	116.00 ± 4.531^***∧▽▽^
11	2-FL(L^b^)+OPN(L^b^)+*DHA*(*L*^b^)	92.667 ± 5.155*
12	2-FL(L^b^)+OPN(M^c^)+*DHA*(*M*^c^)	114.50 ± 6.474^***▽^
13	2-FL(H^a^)+OPN(H^a^)+*DHA*(*H*^a^)	130.167 ± 8.867^***∧∧▽▽▽##^
14	2-FL(LM^d^)+OPN(LM^d^)+*DHA*(*LM*^d^)	107.667 ± 7.315**
15	2-FL(MH^e^)+OPN(MH^e^)+*DHA*(*MH*^e^)	128.50 ± 8.086^***∧∧▽▽▽##^

*p < 0.05 compared with blank control group, **p < 0.01 compared with blank control group, ^***^p < 0.001 compared with blank control group.

^∧^Compared with 2′-FL high-dose group p < 0.05, ^∧∧^compared with 2′-FL high-dose group p < 0.01.

^▽^Compared with OPN high-dose group p < 0.05, ^▽▽^ compared with OPN high-dose group p < 0.01, ^▽▽▽^ compared with OPN high-dose group p < 0.001.

^##^ Compared with DHA high-dose group p < 0.01.

a, high dose; b, low dose; c, medium dose; d, low to medium dose; e, medium to high dose.

### Nutrition promote differentiation of OPCs into mature OLs

To measure the effect of mixed nutrition or nutrition treatment alone on the myelination of OPCs, we assessed the number of MAG-labeled positive cells after treatment for 18 days.

**Figure 2:**
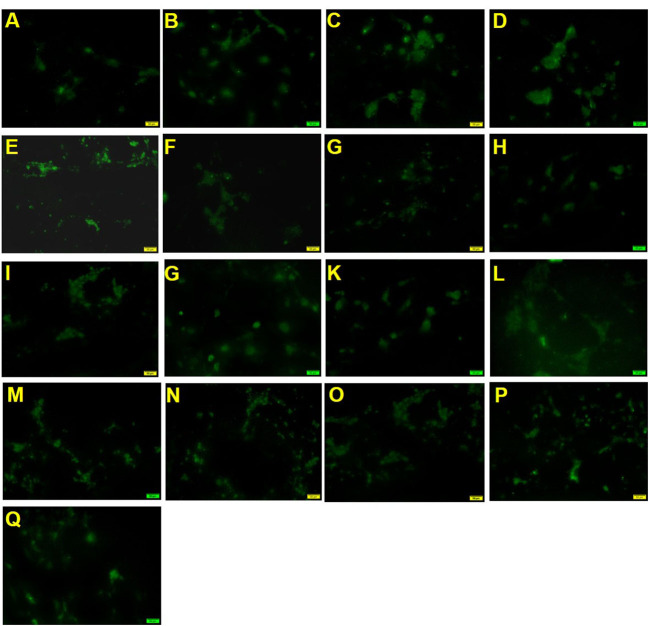
MAG immunostaining of neural primary cells after treatment with 2′-FL and OPN and DHA for 18 days. **(A)**, control group; **(B)**, positive drug; **(C)**, 2′-FL(H); **(D)**, OPN(H); **(E)**, DHA(H); **(F)**, 2′-FL(L)+OPN(L); **(G)**, 2′-FL(L)+OPN(H); **(H)**, 2'-FL(M)+OPN(M); **(I)**, 2′-FL(H)+OPN(L); **(J)**, 2′-FL(H)+OPN(H); **(K)**, 2′-FL(LM)+OPN(LM); **(L)**, 2′-FL(MH) +OPN(MH); **(M)**, 2′-FL(L)+OPN(L)+DHA(L); **(N)**, 2′-FL(L)+OPN(M)+DHA(M); **(O)**, 2′-FL(H)+OPN(H)+DHA(H); **(P)**, 2′-FL(LM)+OPN(LM)+DHA(LM); **(Q)**, 2′-FL(MH)+OPN(MH)+DHA(MH). Scale bar: 50 μm.

As shown in Figure 2 and Table 3, the positive drug olesoxime showed an increase in the number of MAG-positive cells compared to the control group. The 2′-FL OPN and DHA groups (groups 1, 2 and 3) could significantly increase the number of MAG-positive cells, indicating that both components contributed to the differentiation of OPCs into mature OLs. In addition, MAG-positive cells in 2′-FL + OPN middle dose group (group 6), 2′-FL + OPN high dose group (group 8), 2′-FL + OPN middle to high dose group (group 10), and 2′-FL + OPN + DHA high dose (group 13) and 2′-FL + OPN + DHA medium to high dose group (group 15) group were all significantly higher than those of the high dose groups of 2′-FL, OPN and DHA (groups 1, 2 and 3), which suggesting that the combinations of nutrition have synergistic effect to promote differentiation of OPCs into mature OLs.

**Table 3:** Effects of different nutritional compositions containing 2'-FL, OPN and/or DHA on the number of MAG positive cells.

**Groups**	**Nutrition composition**	**Density of MAG positive cells (Mean ±SEM)**
Control	Vehicle	29.333 ± 3.612
Positive	Olesoxime	62.00 ± 5.520***
1	2-FL(H^a^)	51.167 ± 4.624*
2	OPN(H^a^)	52.333 ± 4.944*
3	DHA(H^a^)	47.50 ± 6.999*
4	2-FL(L^b^)+OPN(L^b^)	43.00 ± 5.323*
5	2-FL(L^b^)+OPN(H^a^)	55.167 ± 7.213**
6	2-FL(M^c^)+OPN(M^c^)	60.167 ± 7.441***
7	2-FL(H^a^)+ OPN(L^b^)	56.00 ± 5.422**
8	2-FL(H^a^)+OPN(H^a^)	67.833 ± 7.305^***∧▽#^
9	2-FL(LM^d^)+OPN(LM^d^)	52.833 ± 4.045**
10	2-FL(MH^e^) +OPN(MH^e^)	69.00 ± 7.878^***∧▽#^
11	2-FL(L^b^)+OPN(L^b^)+DHA(L^b^)	46.50 ± 6.402*
12	2-FL(L^b^)+OPN(M^c^)+DHA(M^c^)	59.167 ± 7.596**
13	2-FL(H^a^)+OPN(H^a^)+DHA(H^a^)	73.833 ± 9.250^***∧▽##^
14	2-FL(LM^d^)+OPN(LM^d^)+DHA(LM^d^)	57.167 ± 6.838**
15	2-FL(MH^e^)+OPN(MH^e^)+DHA(MH^e^)	72.00 ± 9.926^***∧▽#^

^*^p < 0.05 compared with blank control group, ^**^p < 0.01 compared with blank control group, ^***^p < 0.001 compared with blank control group.

^∧^ Compared with 2'-FL high-dose group p < 0.05.

^▽^Compared with OPN high-dose group p < 0.05.

^#^ Compared with DHA high-dose group p < 0.05, ^##^ Compared with DHA high-dose group p < 0.01.

a, high dose; b, low dose; c, medium dose; d, low to medium dose; e, medium to high dose.

### Nutrition promotes maturation and myelination of OPCs

To measure the effect of mixed nutrient or nutrient treatment alone on the cell maturation and myelination of OPCs, we assessed the number of positive cells labeled MBP after treatment for 30 days.

**Figure 3:**
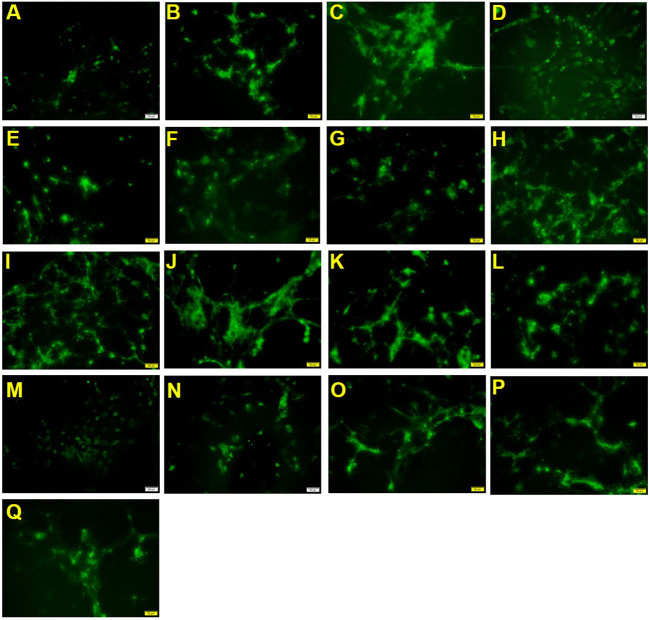
MBP immunostaining of neural primary cells after treatment with 2′-FL and OPN and DHA for 30 days. **(A)**, control group; **(B)**, positive drug; **(C)**, 2′-FL(H); **(D)**, OPN(H); **(E)**, DHA(H); **(F)**, 2′-FL(L)+OPN(L); **(G)**, 2′-FL(L)+OPN(H); **(H)**, 2'-FL(M)+OPN(M); **(I)**, 2′-FL(H)+OPN(L); **(J)**, 2′-FL(H)+OPN(H); **(K)**, 2′-FL(LM)+OPN(LM); **(L)**, 2′-FL(MH) +OPN(MH); **(M)**, 2′-FL(L)+OPN(L)+DHA(L); **(N)**, 2′-FL(L)+OPN(M)+DHA(M); **(O)**, 2′-FL(H)+OPN(H)+DHA(H); **(P)**, 2′-FL(LM)+OPN(LM)+DHA(LM); **(Q)**, 2′-FL(MH)+OPN(MH)+DHA(MH). Scale bar: 50 μm.

According to Figure 3 and Table 4, the positive drug olesoxime increased the number of MBP-positive cells compared with the blank control group. The 2′-FL, OPN and DHA groups (groups 1, 2 and 3) also could significantly increase the number of MBP-positive cells, which suggests that the components contributed to the maturation of OPCs. For mixed nutrition, 2′-FL + OPN high dose group (group 8), 2′-FL + OPN middle to high dose group (group 10), 2′-FL low dose + OPN medium dose + DHA medium dose (group 12), 2′-FL + OPN + DHA high dose (group 13), and 2′-FL + OPN + DHA medium to high dose (group 15) were significantly higher than 2′-FL, OPN or DHA single dose, indicating that these combinations have synergistic effect in maturation and myelination promotion of OPCs.

**Table 4:** Effects of different nutritional compositions containing 2'-FL, OPN and/or DHA on the number of MBP positive cells.

**Groups**	**Nutrition composition**	**Density of MBP positive cells (Mean ±SEM)**
Control	Vehicle	44.333 ± 4.447
Positive	Olesoxime	82.167 ± 6.226***
1	2-FL(H^a^)	79.00 ± 6.846**
2	OPN(H^a^)	73.167 ± 6.215*
3	DHA(H^a^)	79.833 ± 7.507**
4	2-FL(L^b^)+OPN(L^b^)	65.833 ± 8.479*
5	2-FL(L^b^)+OPN(H^a^)	82.333 ± 7.961***
6	2-FL(M^c^)+OPN(M^c^)	97.667 ± 7.365^***▽^
7	2-FL(H^a^)+ OPN(L^b^)	89.50 ± 8.936***
8	2-FL(H^a^)+OPN(H^a^)	128.50 ± 8.221^***∧∧∧▽▽▽###^
9	2-FL(LM^d^)+OPN(LM^d^)	84.667 ± 7.830***
10	2-FL(MH^e^) +OPN(MH^e^)	122.833 ± 8.518^***∧∧∧▽▽▽^
11	2-FL(L^b^)+OPN(L^b^)+DHA(L^b^)	72.667 ± 8.135*
12	2-FL(L^b^)+OPN(M^c^)+DHA(M^c^)	102.00 ± 9.331^***∧▽#^
13	2-FL(H^a^)+OPN(H^a^)+DHA(H^a^)	132.833 ± 10.663^***∧∧∧▽▽▽###^
14	2-FL(LM^d^)+OPN(LM^d^)+DHA(LM^d^)	97.333 ± 5.818^***▽^
15	2-FL(MH^e^)+OPN(MH^e^)+DHA(MH^e^)	135.00 ± 9.913^***∧∧∧▽▽▽###^

^*^p < 0.05 compared with blank control group, ^**^p < 0.01 compared with blank control group, ^***^p < 0.001 compared with blank control group.

^∧^Compared with 2′-FL high-dose group p < 0.05, ^∧∧∧^ compared with 2′-FL high-dose group p < 0.001.

^▽^Compared with OPN high-dose group p < 0.05, ^▽▽▽^Compared with OPN high-dose group p < 0.001.

^#^ Compared with DHA high-dose group p < 0.05, ^###^ Compared with DHA high-dose group p81 < 0.001.

a, high dose; b, low dose; c, medium dose; d, low to medium dose; e, medium to high dose.

Human milk oligosaccharide (HMO) is one of the main components of human milk carbohydrates, which is closely related to the health benefits and nutrition of breastfed infants (21). As the most abundant fucosylated HMO, 2'-FL possesses various beneficial health effects as suppressing pathogen infection, regulating intestinal flora, and boosting immunity, making it has remarkable value in nutrition and medicine (22–25). Additionally, it was verified that the intake of 2′-FL affects the cognitive domain and improves the learning and memory ability of rodents (26). Thus, 2′-FL has lots of beneficial health activities. Due to its various physiological and biological effects, 2′-FL has been assessed and authorized as a new food additive to many foods. OPN is an acidic and highly phosphorylated glycoprotein, and expressed in a variety of tissues including liver, skeletal muscle, brain, and mammary gland (27, 28). Previous studies revealed that OPN is significantly involved in immunity system development and regulation. Evidence indicated that OPN plays a key role in some autoimmune, cancer and cardiovascular disease (29–31). Recently, further researches revealed an important function of OPN involving in the regulation of myelination in central nervous system (32, 33). OPN is relatively resistant to digestion, and orally ingested OPN can be absorbed into the circulatory system (33), therefore, making it plays essential roles in the development in early life and an ideal milk powder additive. DHA is well-known for its effects in intellectual development in early life. Although it plays a key role in the growth and maturation of the infant's brain, DHA cannot be synthesized efficiently in the body (34). During rapid phases of brain growth, large amounts of DHA is needed. Thus, adding DHA to milk powder as an external source has become an inevitable choice when the supply of breast milk is insufficient.

Although 2′-FL, OPN and DHA are important for cognitive development as the milk powder additives, the effect of the three components for neuronal maturation, synaptogenesis, and myelination still little is known. In this study, we evaluated the effect of the three nutrients on promoting the myelination, including the proliferation of OPCs, the differentiation and maturation of OPCs into myelinating OLs, of primary neuronal cells *in vitro*. The results showed that the three components contributed to the proliferation, differentiation, maturation and myelination of OPCs. They have similar effect with the positive nutrient olesoxime in different growth stages of OPCs. When the three components were used in pairs or in combination, these combinations showed a synergistic effect in promotion of OPCs, and the promotion effect was obviously stronger than that of the nutrition treatment alone or even the positive drug olesoxime.

In short, the current study described an *in vitro* model to test the effects of 2'-FL, OPN, DHA and a nutrient blend consisting of the three nutrients on increasing OPC maturation as well as OL myelination. This most promoting effect was driven by a combined net of nutrient blend, and individual nutrients did not exhibit the same positive effect on myelination, which showing the important of nutrients composition in infant formula. For excise mechanism, the results would need to be further verified with an *in vivo* experiment (35). Besides, studies of gene or protein expressions in OLs in response to the various individual nutrients as well as the nutrient blend are required to identify the signal pathways involved in the myelination related effects in the future (36).

## Conclusions

As the most abundant constituents in human breast milk or infant formula, 2′-FL OPN and DHA have numerous beneficial health effects, especially during the intellectual development of infant. Our work elucidates a pro-myelinative effect of 2′-FL, OPN and DHA with neural primary cell *in vitro*. The potential mechanism of the nutrients included induction of oligodendrocyte precursor proliferation, differentiation, maturation and myelination *via* which this effect might be mediated. The study also showed that when the three nutrient components were used in in pair or in combination, their effect on promoting OPCs myelination was significantly better than that of the individual components. The results of this experiment clarified the mechanism of 2′-FL OPN and DHA to promote cognitive development, and provided a solid experimental basis for further optimization of infant formula.

## Data availability statement

The original contributions presented in the study are included in the article/supplementary material, further inquiries can be directed to the corresponding authors.

## Ethics statement

The animal study was reviewed and approved by the Laboratory Animal Center of Peking University Health Science Center.

## Author contributions

QX, YZ, MG, and QZ designed the study and manuscript writing. QX, JZ, and DC did the laboratory work in the expression and statistical analysis. JZ, DC, and QZ contributed to data analysis, interpretation, and the revision of articles critically for important intellectual content. All the authors read and approved the manuscript.

## Funding

This research was funded by Technology Major Special Project of Heilongjiang Province: Dairy Products and Meat Processing (No. 2020ZX07B01), Beijing Municipal Excellent Talents Foundation (2018400685627G341), Fengtai Nova Program (KJXX201904), and Beijing Postdoctoral Research Foundation.

## Conflict of interest

Authors QX and DC were employed by the company Heilongjiang Feihe Dairy Co., Ltd. The remaining authors declare that the research was conducted in the absence of any commercial or financial relationships that could be construed as a potential conflict of interest.

## Publisher's note

All claims expressed in this article are solely those of the authors and do not necessarily represent those of their affiliated organizations, or those of the publisher, the editors and the reviewers. Any product that may be evaluated in this article, or claim that may be made by its manufacturer, is not guaranteed or endorsed by the publisher.
